# Severe COVID-19-Induced Hemophagocytic Lymphohistiocytosis

**DOI:** 10.7759/cureus.34022

**Published:** 2023-01-20

**Authors:** Shahkar Khan, Taqi A Rizvi, Waleed Sadiq, Saud Bin Abdul Sattar, Rabih Maroun

**Affiliations:** 1 Internal Medicine, Staten Island University Hospital, New York City, USA; 2 Pulmonary and Critical Care Medicine, Staten Island University Hospital, New York City, USA

**Keywords:** coronavirus disease 2019, cytokine storm syndrome, multiorgan system failure, acute hypoxic respiratory failure, covid 19, hemophagocytic lymphohistiocytosis (hlh)

## Abstract

We reported a case of secondary hemophagocytic lymphohistiocytosis (HLH), a rare and life-threatening condition, which was suspected to have been triggered by a severe case of coronavirus disease 2019 (COVID-19). A 50-year-old man with a past medical history of ulcerative colitis with recent pancolitis status post colectomy and ileostomy two weeks before presentation presented to the emergency department with one week of subjective fevers, weakness, watery diarrhea, and decreased oral intake. A CT scan showed fluid in the rectum and post-surgical changes from his recent colectomy along with diffuse reticulonodular opacities of the lungs. His COVID-19 reverse transcriptase-polymerase chain reaction (RT-PCR) test was positive. Over the subsequent days, the patient's condition worsened as he developed worsening acute hypoxic respiratory failure with diffuse lymphadenopathy, splenomegaly, worsening cytopenias, and increased ferritin of >100,000 ng/ml on hospital day six. Hematology oncology was consulted and he was started on empiric steroid therapy followed by etoposide. However, his condition continued to worsen, and eventually, the patient passed away on hospital day eight.

## Introduction

Hemophagocytic lymphohistiocytosis (HLH) is a rare life-threatening condition that is a result of a hyper-inflammatory state. Due to its high mortality rate, prompt diagnosis is of the utmost importance [1,2]. Diagnosis of HLH can be established using the Histiocyte Society criteria (HLH-2004) [3]. Here, we report a case of secondary HLH, which was suspected to have been triggered by severe coronavirus disease 2019 (COVID-19).

## Case presentation

A 50-year-old man presented to the emergency department with one week of subjective fevers, weakness, watery diarrhea seen through the ileostomy bag, and decreased oral intake. He had a past medical history of ulcerative colitis with recent pancolitis status post colectomy and ileostomy two weeks before presentation. He was taking acetaminophen occasionally for aches and pains. The patient did not report shortness of breath, chest pain, cough, dizziness, numbness, tingling, recent sick contacts, or recent travel. He was up to date with his COVID-19 vaccination.

On presentation, his vitals showed a heart rate of 149 beats per minute, blood pressure of 86/59 mmHg, respiratory rate of 17 breaths per minute, a temperature of 97 degrees Fahrenheit, and oxygen saturation of 100% on room air. He also underwent a COVID-19 nasal swab polymerase chain reaction (PCR) test, which was positive. Significant results for the patient's initial blood work are listed in Table 1.

**Table 1:** Pertinent lab values on admission

Pertinent Lab	Value	Reference Range
White blood cell count	3.4 K/uL	4.80-10.80 K/ul
Hemoglobin	9.8 g/dl	14.0-18.0 g/dl
Platelet count	139 K/ul	130-400 k/ul
Sodium (serum)	121 mmol/L	135-146 mmol/L
Osmolality (serum)	255 mos/Kg	275-300 mos/Kg
D-dimer	6492 ng/ml DDU	0-230 ng/ml DDU
Fibrinogen	700 mg/dL	204-570 mg/dl
Lactate dehydrogenase	1034 U/L	50-242 U/L
C-reactive protein	267.7 mg/L	<= 4.0 mg/L
Lactate	1.4 mmol/L	0.7-2.0 mmol/L
Alkaline phosphatase	775 U/L	30-115 U/L
Aspartate aminotransferase	299 U/L	0-41 U/L
Serum bicarbonate	16 mmol/L	17-32 mmol/L

**Figure 1:**
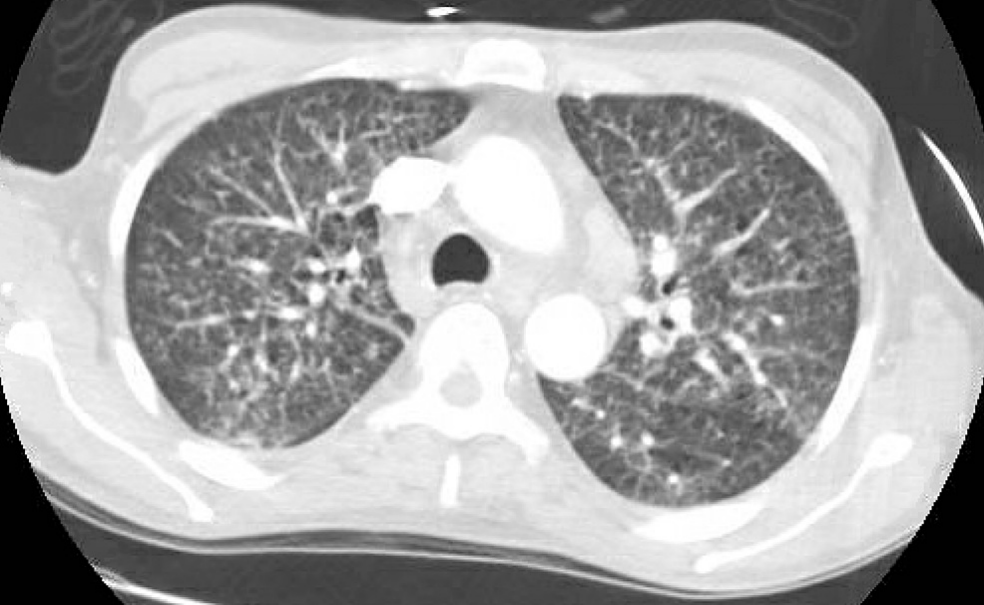
Reticulonodular opacities shown in CT of the chest

**Figure 2:**
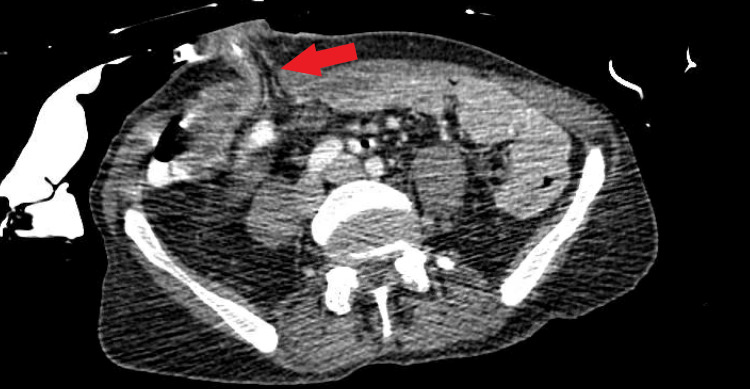
Ileostomy shown in CT of the abdomen and pelvis

**Figure 3:**
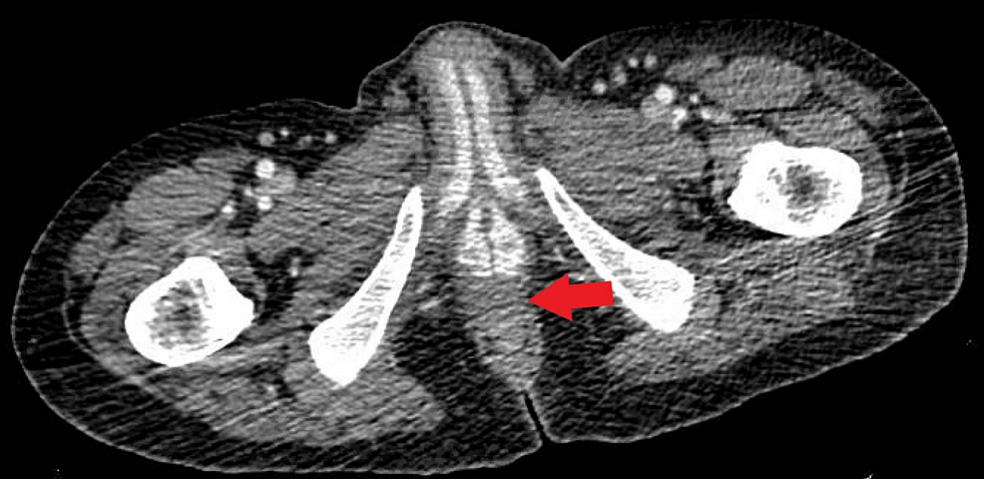
Perirectal fluid shown in CT of the abdomen and pelvis

Computerized tomography with oral contrast of the abdomen and pelvis showed reticular nodular opacities in the lung (Figure 1), evidence of colectomy with ileostomy (Figure 2) in the right lower quadrant, fluid in the rectum (Figure 3), and an enlarged spleen.

**Figure 4:**
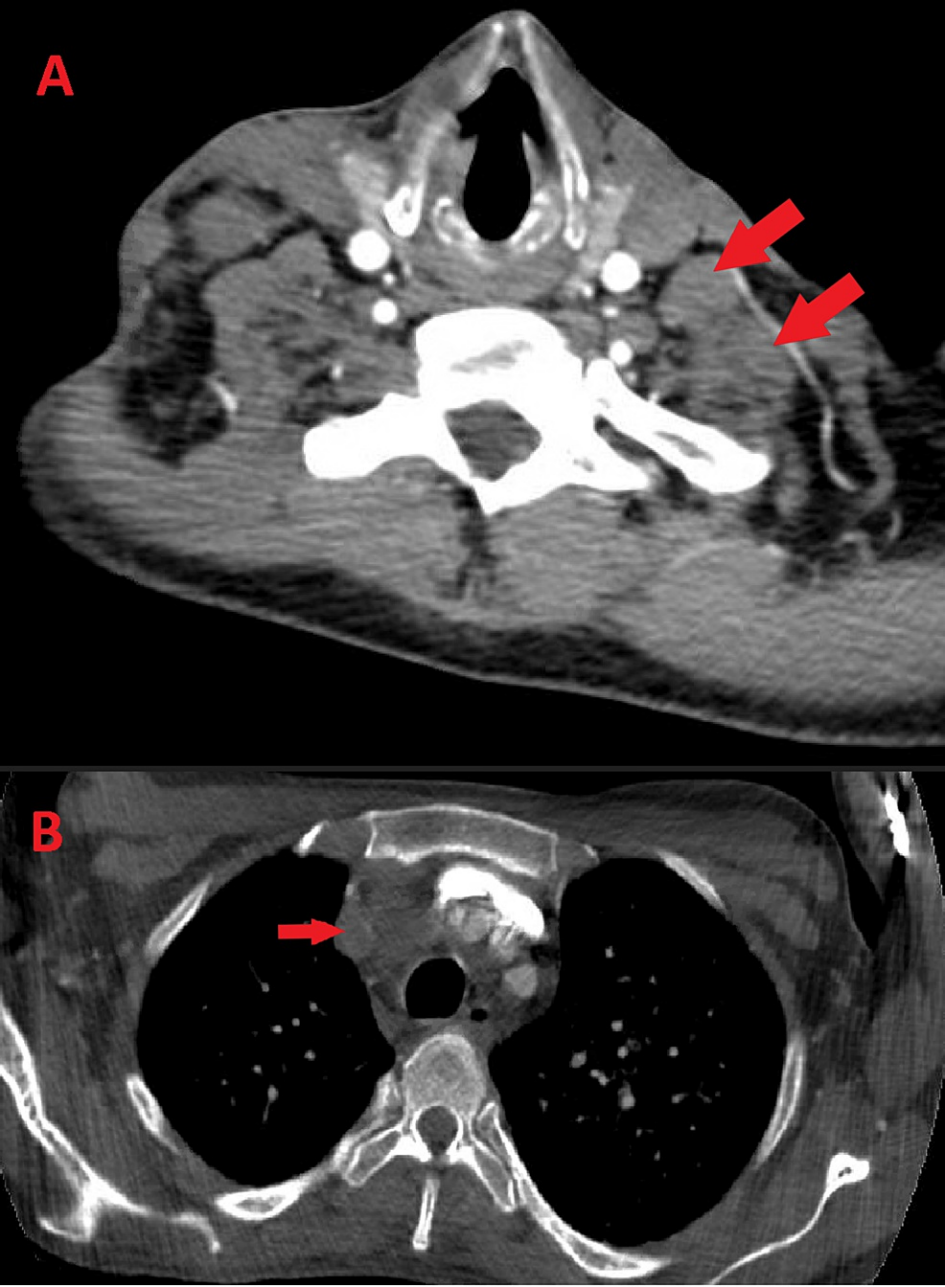
CT images of the chest showing (A) supraclavicular lymphadenopathy and (B) mediastinal lymphadenopathy

Due to sinus tachycardia on electrocardiogram and elevated d-dimer, pulmonary embolism was also suspected, and a computerized tomography pulmonary embolism protocol was performed, which did not reveal any clots in the pulmonary vasculature; however, diffuse reticulonodular interstitial thickening with supraclavicular and mediastinal lymphadenopathy was observed (Figure 4).

The patient received fluids, intravenous vancomycin, intravenous cefepime, intravenous metronidazole, and oral acetaminophen. He was subsequently admitted for the workup and management of a suspected unknown infectious etiology. The patient, the following day, became increasingly septic and had tachycardia. Ferritin increased from 46718 on admission to >100,000 ng/ml on hospital day six, and D-dimer increased from 6492 on admission to 11188 ng/ml DDU on hospital day six. In addition, he had persistent fevers and worsening cytopenia.

All infectious workups at this point came back negative including blood cultures, urine cultures, blood smears for different pathogens, stool test for *Clostridium difficile*, gastrointestinal pathogen panel, and HIV. Blood tests for Epstein-Barr virus (EBV) and cytomegalovirus (CMV) only revealed a past EBV infection.

The patient was referred to the critical care team and was moved to the intensive care unit. Due to the high suspicion of HLH, a bone marrow biopsy was performed, which showed increased natural killer cells but there was no evidence of hemophagocytic histiocytes; however, his soluble CD25 was elevated at 49699.3 pg/ml. Thus, he fulfilled five out of eight HLH criteria [3], which were splenomegaly, pancytopenia, elevated soluble CD25, elevated serum ferritin, and fever of more than or equal to 38.5 degrees Celsius. He was then started on intravenous steroids due to suspected HLH. The patient was also treated with remdesivir for COVID-19.

The patient started to develop acute hypoxic respiratory failure. His lactate also started rising and an X-ray of the chest revealed asymmetrical bilateral consolidations and acute respiratory distress syndrome (ARDS) was suspected. Oxygen saturation kept on dropping despite the patient being placed on bilevel-positive airway pressure and the patient was eventually intubated and placed on mechanical ventilation. The patient was also becoming increasingly hypotensive during this time requiring intravenous vasopressors and eventually required three different intravenous vasopressors to maintain hemodynamic stability. The patient was also started on intravenous etoposide for HLH but clinically started to decline rapidly. The primary team had a discussion with the patient’s spouse and due to the overall prognosis being poor, she decided to make the patient comfort measures only with palliative extubation, and the patient expired shortly after.

## Discussion

HLH is described as a syndrome of a hyper-inflammatory state, which is caused by the defective function of natural killer cells and macrophages [1,2]. It is a rare condition and can be life-threatening. It is reported to have all-cause mortality ranging from 41% to 75% [3]. Diagnosis of HLH is based on the HLH-2004 trial, which includes pathologic mutations of PRF1, UNC13D, Munc18-2, Rab27a, STX11, SH2D1A, or BIRC4 HLH, or if five of the eight clinical presentations are fulfilled, which includes fever, splenomegaly, cytopenia of two or more lineages, hyperferritinemia, hypofibrinogenemia or hypertriglyceridemia, elevated soluble CD25, hemophagocytosis, and reduced or absent natural killer cytotoxicity seen on biopsy [4].

HLH can be further classified into two groups: primary HLH and secondary HLH. Primary HLH is caused by a defective mutation and secondary HLH is usually in patients without a known culprit mutation who are exposed to a trigger that causes HLH [5]. The known triggers include viruses, bacteria, fungi, parasites, and autoimmune diseases among others. The most known pathogenic triggers are viruses most notably EBV and CMV [6]. The most common autoimmune diseases that cause HLH are systemic juvenile idiopathic arthritis, followed by systemic lupus erythematosus, Kawasaki disease, and juvenile dermatomyositis [7].

COVID-19 presents in a wide variety of ways from asymptomatic disease to cytokine storm with multi-organ failure and death. There are multiple clinical similarities between severe COVID-19 with cytokine storm and HLH; these include ARDS, renal failure, fevers, and cytopenia [8]. The occurrences of thrombocytopenia and anemia in COVID-19 patients have been reported at 24% and 59%, respectively [9]. Among the biochemical abnormalities of HLH the typical triad usually includes hypofibrinogenemia, hypertriglyceridemia, and hyperferritinemia. In terms of fibrinogen, COVID-19 patients usually have hyperfibrinogenemia secondary to systemic inflammation but they can develop hypofibrinogenemia in more advanced diseases likely secondary to HLH or disseminated intravascular coagulation (DIC) [10]. This is evidenced by a study that enrolled 183 patients with COVID-19 pneumonia in which, hypofibrinogenemia was noticed in 28.6% of the non-survivors and the fibrinogen level was significantly lower in non-survivors vs survivors [11]. The occurrence of hyperferritinemia is well-documented in both COVID-19 and HLH [4,9]. In terms of triglyceride levels, there was no significant difference seen between severe and non-severe cases of COVID-19 [12]. The occurrence of hypertriglyceridemia has been reported at around 9% in patients with COVID-19 [9]. Tocilizumab-induced hypertriglyceridemia should always be entertained when investigating the HLH criteria in patients with severe COVID-19 [13,14]. There are multiple similarities between severe COVID-19 and secondary HLH. There have been other reported cases as well [15] Therefore, in COVID-19 patients who have worsening widespread inflammation with multiorgan dysfunction including renal failure and ARDS, HLH criteria should be investigated as prompt identification and treatment may improve outcomes [16].

The HLH-94 protocol provides a foundation for the initial treatment of HLH patients and, so far, remains the standard of care [16,17]. The protocol includes the administration of immunosuppressive and cytotoxic agents and intrathecal thecal methotrexate and hematopoietic stem cell transplantation (HSCT) for those who qualify. This has resulted in survival benefits in patients with HLH [16].

## Conclusions

The timely diagnosis of HLH is of the utmost importance due to the associated high mortality rate and should be high on the differential diagnosis list if suspected. Secondary HLH can be difficult to manage if the triggering etiology is difficult to decipher. We propose that in COVID-19 patients with worsening clinical status and cytokine, storm HLH should be suspected. These patients need to have an immediate evaluation for HLH criteria as any delay can substantially worsen outcomes for patients.
